# Results of an Innovative Program for Surveillance, Prophylaxis, and Treatment of Infectious Complications Following Allogeneic Stem Cell Transplantation in Hematological Malignancies (BATMO Protocol)

**DOI:** 10.3389/fonc.2022.874117

**Published:** 2022-06-17

**Authors:** Michele Malagola, Alessandro Turra, Liana Signorini, Silvia Corbellini, Nicola Polverelli, Lorenzo Masina, Giovanni Del Fabro, Silvia Lorenzotti, Benedetta Fumarola, Mirko Farina, Enrico Morello, Vera Radici, Eugenia Accorsi Buttini, Federica Colnaghi, Simona Bernardi, Federica Re, Arnaldo Caruso, Francesco Castelli, Domenico Russo

**Affiliations:** ^1^Chair of Hematology, Bone Marrow Transplant Unit, ASST-Spedali Civili Brescia, Depatment of Clinical and Experimental Sciences University of Brescia, Brescia, Italy; ^2^Department of Infectious and Tropical Diseases, Azienda Socio Sanitaria Territoriale (ASST) Spedali Civili Hospital, University of Brescia, Brescia, Italy; ^3^Department of Molecular and Translational Medicine, Section of Microbiology and Virology, University of Brescia Medical School, Brescia, Italy; ^4^Centro di Ricerca Ematologico – Associazione Italiana per la Lotta alle Leucemie, Linfomi e Mieloma (AIL), Azienda Socio Sanitaria Territoriale (ASST) Spedali Civili di Brecia, Brescia, Italy

**Keywords:** prophylaxis, fungal infections, viral infections, multi-drug resistance, bacterial infections

## Abstract

**Background:**

Infectious complications are a significant cause of morbidity and mortality in patients undergoing allogeneic haematopoietic stem cell transplantation (Allo-SCT). The BATMO (Best-Antimicrobial-Therapy-TMO) is an innovative program for infection prevention and management and has been used in our centre since 2019. The specific features of the BATMO protocol regard both prophylaxis during neutropenia (abandonment of fluoroquinolone, posaconazole use in high-risk patients, aerosolized liposomal amphotericin B use until engraftment or a need for antifungal treatment, and letermovir use in CMV-positive recipients from day 0 to day +100) and therapy (empirical antibiotics based on patient clinical history and colonization, new antibiotics used in second-line according to antibiogram with the exception of carbapenemase-producing *K pneumoniae* for which the use in first-line therapy is chosen).

**Methods:**

Data on the infectious complications of 116 transplant patients before BATMO protocol (Cohort A; 2016 - 2018) were compared to those of 84 transplant patients following the introduction of the BATMO protocol (Cohort B; 2019 - 2021). The clinical and transplant characteristics of the 2 Cohorts were comparable, even though patients in Cohort B were at a higher risk of developing bacterial, fungal, and CMV infections, due to a significantly higher proportion of myeloablative regimens and haploidentical donors.

**Results:**

No change in the incidence of infections with organ localization was observed between the two Cohorts. A significant reduction in *Clostridioides difficile* infections by day +100 was observed in Cohort B (47% vs. 15%; p=0.04). At day +30, a higher incidence of Gram-negative bloodstream infections (BSIs) was observed in Cohort B (12% vs. 23%; p=0.05). By day +100 and between days +100 and +180, the incidence of BSIs and of the various etiological agents, the mortality from Gram-negative bacteria, and the incidence of invasive fungal infections were not different in the two Cohorts. The incidence of CMV reactivations by day +100 dropped drastically in patients of Cohort B, following letermovir registration (51% vs. 15%; p=0.00001).

**Discussion:**

The results of this study suggest that the BATMO program is safe. In particular, the choice to avoid prophylaxis with fluoroquinolone was associated with an increase in Gram-negative BSIs by day +30, but this did not translate into higher levels of mortality. Moreover, this strategy was associated with a significant reduction of *Clostridiodes difficile* infections. The efficacy of anti-CMV prophylaxis with letermovir was confirmed by a significant reduction in CMV reactivations. Even though patients in Cohort B were at higher risk of developing fungal infections (more haploidentical transplants with more myeloablative regimens), the extensive use of posaconazole for prophylaxis balanced this risk, and no increase in the incidence of fungal-associated complications was observed.

## Introduction

Bacterial, fungal, and viral infections frequently complicate the clinical course of allogeneic stem cell transplantation (Allo-SCT) (1). The time at which infections occur after transplantation depends on several factors, the most significant of which are the degree and duration of neutropenia and the severity of immunosuppression (2). Overall, infectious complications may occur up to one year following allo-SCT, and this time period becomes longer in case of chronic graft versus host disease (cGVHD) (1, 2).

Historically, the backbone of anti-infectious prophylaxis for allotransplanted patients is based on fluoroquinolone and fluconazole, during neutropenia, together with acyclovir and cotrimoxazole until an adequate immune reconstitution (e.g., CD4+ cells higher than 200/mcl) (3–6). The rationale for this approach is the prevention of bacterial, viral, and fungal infections (namely sustained by Gram-negative species, candida, herpes simplex virus, and *pneumocystis j*). The optimal management of infectious complications in patients submitted to allo-SCT includes colonization monitoring through mucosal swabs, blood cultures in the case of fever higher than 38°C, and the prompt introduction of empirical antimicrobial therapy. The latter needs to be implemented and adapted based on the clinical course, the results of the cultures, and of instrumental assessment (e.g., chest x-ray and thorax CT scan) (7).

Since 2007, when the first allo-SCT was performed in our centre, we have followed the above approach. In particular, in the case of fever above 38°C during neutropenia, the first-line antibiotic therapy consisted of ceftazidime +/- amikacin and/or vancomycin based on patient clinical conditions (7).

Over the last 10 years, there has been significant developments in the transplant platforms, due to several factors (8): the increase in age limits for transplant eligibility (now up to 75 years) (9), the use of alternative donors (namely haploidentical) (10), the use of *in vivo* T-cell depletion (e.g., T anti-lymophocyte globulin - ATLG) (11), the use of post-transplant cyclophosphamide (10) and, finally, the introduction of new drugs in the conditioning regimens (e.g., treosulfan) (12). These factors, together with the selective anti-microbial pressure induced by the forementioned approach, significantly modified the spectrum of infectious complications over time. In particular, bacterial infections sustained by fluoroquinolone-resistant species (namely *Enterobacterales*), fungal infections sustained by *Aspergillus spp*, and viral infections sustained by herpes viruses other than CMV (e.g., HHV-6), have been more frequently observed in patients transplanted in recent years (1).

With this background, and following revision of local epidemiology, since January 2019 we have modified the management of infectious complications following a novel approach, specifically designed together with our colleagues in the Infectious Disease and Microbiology units of our hospital. This approach has been named BATMO (Best Antimicrobial Therapy in TMO - Trapianto di Midollo Osseo). In this paper, we report the results of this approach, employed over the last 2 years, in terms of incidence and distribution of bacterial, fungal, and viral complications, and in comparison with dataobserved between 2016 and 2018.

## Patients and Methods

The study included 200 patients who were submitted to allo-SCT from January 2016 to January 2021: in particular, 116 patients underwent transplant between 2016 and 2018 (Cohort A) and 84 underwent transplant between 2019 and 2021 (Cohort B). These 200 patients represent all of the patients consecutively submitted to allo-SCT between 2016 and 2021 at our institution, and this series is consecutive to the previous series analysed and published in 2017 (7). Infectious complications of Cohort B were managed according to the BATMO program. The aim of this retrospective study was to assess the efficacy of the BATMO program in terms of OS. Moreover, this study aimed to assess how BATMO modified the incidence of bacterial, fungal, and viral infections with organ localization, BSIs, and viral reactivations, with a temporal difference in early (until +30 and +100 days from allo-SCT) and late infections (from day +100 to day +180), with respect to the control Cohort A (2016–2018). Clinical data were collected from the computerized database, in order to include all the transplant patients’ information. All the patients have been registered in the European PROMISE database for transplant activity and gave their consent for the collection of clinical and transplant data.

Notably, pre-transplant prophylaxis and treatment of bacterial and fungal infections have been conducted in the clinical unit of hematology of our hospital and did not significantly change in the last 15 years. Briefly, prophylaxis consisted of levofloxacin in case of expected neutropenia longer than 7 days and posaconazole and itraconazole for acute leukemias during induction and consolidation, respectively. First-line antibiotics in case of fever during neutropenia consisted of ceftazidime and amikacin with or without glycopeptide in the case of mucositis or suspected Gram-positive infection.

### Best Antimicrobical Therapy TMO (BATMO)

The BATMO program was designed in collaboration with our colleagues from the Infectious Diseases and Microbiology units, according to revised data on local epidemiology and the outcome of patients submitted to allo-SCT from 2016 to 2021 (**Table 1**). The diagnostic approach for bacterial, fungal, and viral infections remained unchanged between the two time periods.

**Table 1:** Characteristics of BATMO protocol for prophylaxis and treatment of infections in allotransplanted patients.

Prophylaxis	Therapy
Antibacterial prophylaxis with levofloxacin is suspended during the phase of neutropenia. Antifungal prophylaxis with fluconazole is reserved only for low-risk patients according to the score proposed by Stanzani et al. (13), while in high-risk patients the use of posaconazole is introduced. Aerosolized liposomal amphotericin B according to Rijnders et al. 2.5 ml/daily of a 5mg/ml solution in the first week, thereafter 2.5 ml for 2 consecutive days every week until engraftment or need for antifungal treatment (14) In all CMV seropositive patients, the use of letermovir is introduced, up to day +100 from allo-SCT.	The use of antibiotics in empirical therapy is based on the patient’s clinical history and its possible colonization by MDRO. First-line therapy consists in the use of piperacillin-tazobactam or ceftazidime or cefepime, possibly in association with a glycopeptide in case of suspected infection with Gram positive bacteria. Second-line drugs, such as carbapenems, and new antibiotics, such as ceftazolan-tazobactam or ceftazidime-avibactam, should be used based on the antibiogram data, except in patients colonized by *Klebsiella pneumoniae* KPC (first-line therapy)

Compared to the historical approach, the main features of this program concern both prophylaxis and the therapeutic management of infectious complications.

Firstly, antibacterial prophylaxis with levofloxacin, during the phase of neutropenia was suspended. Secondly, antifungal prophylaxis with fluconazole was reserved only for patients at low risk of invasive fungal disease (IFD) according to the score proposed by Stanzani and Colleagues (13). Conversely, in high-risk patients, posaconazole was introduced. Moreover, for all patients, fungal prophylaxis was supplemented with aerosolized liposomal amphotericin B according to Rijnders et al. (2.5 ml/daily of a 5mg/ml solution in the first week, followed by 2.5 ml for 2 consecutive days every week until engraftment or a need for antifungal treatment) (14). Thirdly, following the results of the registrative study, all CMV IgG positive patients received letermovir prophylaxis from day 0 to day +100 (15). Acyclovir from the day of SCT and contrimoxazole from the day of engraftment remained the standard prophylaxis for herpes simplex and *pneumocystis j*, until immunological reconstitution (CD4+ cells grater than 200/mmc).

The monitoring of viral reactivations was comparable in the two Cohorts, and consisted of CMV, HHV6, and EBV DNAemia assessment weekly from the start of conditioning up to day +90, and then every 2 weeks until day +180. For other viruses (e.g., respiratory viruses including SARS CoV2) the monitoring was performed based on symptoms (e.g., cough, fever, cold),. Toxoplasma monitoring with molecular biology was performed only in the case of symptoms potentially related to this infection. Focusing on fungal infections, galactomannan antigen was monitored on peripheral blood in both Cohorts weekly, from the day of SCT until day +90, and then every 2 weeks until day +180. This protocol of viral and fungal reactivations monitoring was intensified in high-risk patients (e.g., aGVHD under steroids).

With regards to the therapeutic management of infectious complications, the BATMO program has highlighted the importance of the rational use of antibiotics in empirical therapy based on patient’s clinical history and colonization by multi-drug resistant organisms (MDRO). In the case of suspected bacterial infection, the first-line therapy consisted of piperacillin/tazobactam or ceftazidime or cefepime, in association with a glycopeptide in the case of possible infection with Gram-positive bacteria. Second-line drugs, such as carbapenems, and new antibiotics, such as ceftazolan-tazobactam or ceftazidime-avibactam, have been used on the basis of antibiogram, with the exception of patients colonized by carbapenemase-producing *K pneumoniae* (KPC), for whom they represent the first line of therapy.

### Conditioning Regimens and GVHD Prophylaxis

The conditioning regimens used in the two Cohorts were comparable, as our internal guidelines did not significantly change over the two time periods. Briefly, myelo-ablative conditioning regimens (MAC) included intravenous busulfan (total dose 12.8 mg/Kg) or treosulfan (total dose 42 mg/sqm) combined with fludarabine or thiotepa (total dose 10 mg/Kg) in combination with busulfan (total dose 9.6 mg/Kg) and fludarabine. Myeloablative total body irradiation (TBI) was 1000-1200 cGy in five or six fractions (200 cGy each) with fludarabine. Reduced-intensity conditioning regimens (RIC) included intravenous busulfan (total dose 6.4 – 9.6 mg/Kg) or treosulfan (total dose 30 mg/sqm) combined with fludarabine or thiotepa (total dose 10 mg/Kg) in combination with ciclophosphamide (total dose 100 mg/Kg) and fludarabine.

Similarly, GVHD prophylaxis was homogeneous over the two time periods. The most frequently used standard prophylaxis included cyclosporine, methotrexate, and ATLG. ATLG was omitted only in the case of patients submitted to allo-SCT from a matched sibling donor, using bone marrow as a stem cell source. Patients submitted to haploidentical SCT received the conventional combination of cyclosporine, mycophenolate, and post-transplant cyclophosphamide.

### Statistical Analysis

All variables have been summarized as median and range or percentage; differences among subgroups have been detected according to the t-test and Chi-square test, respectively. 100-days Landmark overall survival (OS) has been analysed using the Kaplan-Meier method, from the date of transplant to the date of death or last follow-up. The log-rank test was carried out to detect significant differences among transplant Cohorts. All p values <0.05 were considered statistically significant. Statistical analysis was performed with EZR software (v. 1.53) (16).

## Results

### Patients’ and Transplant Characteristics

**Table 2** reports the most important clinical and transplant characteristics of patients including the Cohort A (n=116 patients transplanted between 2016 and 2018) and Cohort B (n=84 patients transplanted between 2019 and 2021). Significant differences between the two Cohorts were: a higher prevalence of acute leukemias and myelodisplastic syndromes in Cohort A (72% vs. 57%; p=0.02) and of multiple myeloma in Cohort B (4% vs. 14%, p=0.01). All the other clinical characteristics are well balanced between the two groups, including the rate of CMV IgG positive cases (74% in Cohort A and 78% in Cohort B). Notably, we found: higher use of MAC regimens in Cohort B (48% vs. 62%, p=0.05), and more frequent use of matched sibling donors (MSD) in Cohort A (33% vs. 20%, p=0.05), which is counterbalanced by more frequent use of haploidentical (Haplo) donors in Cohort B (18% vs. 30%, p=0.05).

**Table 2:** Clinical and transplant characteristics of the 200 allotransplanted patients included in this analysis.

Variable	Total (n = 200)		Cohort A (2016-2018) (n = 116)		Cohort B (2019-2021) (n = 84)		P-value
	**N°**	**%**	**N°**	**%**	**N°**	**%**	
**Sex**							
Male	119	60	64	55	55	66	0.14
Female	81	40	52	45	29	34	
**Age at allo-SCT**							
Median (years)	55		56		53		0.94
Range (years)	16-73		16-71		21-73		
**CMV serostatus**							
IgG positive	150	75	85	73	65	77	0,51
**Disease**							
ALs and MDSs	132	66	84	72	48	57	**0.02**
Lymphoproliferative disorders	23	12	13	11	10	12	0.88
CMDs	22	11	12	10	10	12	0.73
MM	17	8	5	4	12	14	**0.01**
Others	6	3	2	2	4	5	0.21
**Disease phase**							
Frontline	2	1	0		2	2	–
1st CR	59	29	34	29	25	30	0.94
> 1st CR	139	70	82	71	57	68	0.67
**Conditioning regimens**							
MAC	108	54	56	48	52	62	**0.05**
RIC	92	46	60	52	32	38	
**Source of SCs**							
BM	47	23.5	32	28	15	18	0.11
PBSC	152	76	83	71	69	82	0.08
UCB	1	0.5	1	1	0		
**Donor type**							
MSD	55	28	38	33	17	20	**0.05**
MUD	98	49	56	48	42	50	0.81
Haploidentical	46	23	21	18	25	30	**0.05**
Cord blood	1	0,5	1	1	0	0	–
**GVHD prophylaxis**							
Including ATLG	133	66	80	69	53	63	0.39
Non including ATLG	14	7	10	9	4	5	0.29
Including CTX post SCT	46	23	21	18	25	30	**0.05**
Other	7	3	5	4	2	2	0,53

ALs, acute leukemias; MDSs, myelodysplastic syndromes; CMDs, chronic myeloprolipherative disorders; MM, multiple myeloma; CR, complete remission; MAC, myeloablative conditionig; RIC, reduced-intensity conditioning; SCs, stem cells; BM, bone marrow; PBSC, peripheral blood stem cells; UCB, umbilical cord blood; MSD, matched sibling donor; MUD, matched unrelated donor; ATLG, T anti-lymophocyte globulin; CTX, post-transplant cyclophosphamide.

Bold values regard the statistical significance (when below 0.05).

The overall incidence of aGVHD was 51% (59/116) and 46% (39/84) in Cohort A and B, respectively (p=0.53). Grade II-IV aGVHD incidence was 34% (40/116) and 33% (28/84) (p=0.86) and grade III-IV aGVHD incidence was 13% (15/116) and 15% (7/84) (p=0.30) in Cohort A and B, respectively. The incidence of severe cGVHD was 3% (3/116) and 4% (3/84) (p=0.68) in Cohort A and B, respectively.

### Pre-Transplant Colonization and Distribution of Infectious Events

**Table 3** reports the distribution of pre-transplant colonization and infectious complications in the 200 patients of this series at days +30, +100, and between days +100 and +180.

**Table 3:** Distribution of pre-transplant colonization and infectious complications in the 200 allotransplanted patients of this analysis.

	Total (n = 200)		Cohort A (2016-2018) (n = 116)		Cohort B (2019-2021) (n = 84)		P-value
	N°	%	N°	%	N°	%	
**Pre-transplant colonization (rectal swab)**	38	19	13	11	22	26	**0,005**
ESBL+	29	14	10	77	19	76	0,94
CRE	4	2	3	23	1	4	0,06
VRE	1	0,5	0	0	1	0	–
**Day +30**							
Patients with at least 1 event	137	68	70	60	67	80	**0.003**
**Infectious complications**	260	–	118	–	142	–	–
With organ localization	113	43	50	42	63	44	0,69
BSIs	79	30	37	31	42	29	0,76
Viral reactivations	68	26	31	26	37	26	0,96
**Day +100**							
Patients with at least 1 event	168	84	95	82	73	87	0.34
**Infectious complications**	493	–	286	–	207	–	–
With organ localization	207	42	116	40	91	44	0,45
BSIs	119	24	65	23	54	26	0,34
Viral reactivations	167	34	105	37	62	30	0,12
**Days +100/+180**							
Patients with at least 1 event	66	33	43	37	23	27	0.15
**Infectious complications**	120	–	67	–	53	–	–
With organ localization	49	41	28	42	21	40	0,81
BSIs	18	15	12	18	6	11	0,32
Viral reactivations	53	44	27	40	26	49	0,34

BSIs, bloodstream infections; ESBL+, extended spectrum beta-lactamases producers; CRE, Carbapenem-Resistant Entrobactriaceae; VRE, Vancomycin Resistant Enterococci.

Bold values regard the statistical significance (when below 0.05).

The rate of pre-transplant colonization was 11% (13/116 patients) and 26% (22/84 patients) in Cohort A and B, respectively (p=0.05). In both Cohorts, most of these colonizations were sustained by extended spectrum beta-lactamases producers Gram-negative bacteria (ESBL+), whereas patients colonized by carbapenem-resistant *Enterobacteriaceae* (CRE) were three in Cohort A and one in Cohort B. According to the antibiograms, these four CRE were defined as MDRO.

On day +30, 260 infectious complications had been registered (118 in Cohort A and 142 in Cohort B). Overall, 70/116 patients (60%) and 67/84 patients (80%) in Cohort A and B, respectively, experienced at least one event (p=0,003). In Cohort A and Cohort B the distribution of infections with organ localization (including fungal infections), BSIs and viral reactivations was: 42% vs. 44% (p=0.69), 31% vs. 29% (p=0.76), and 26% vs. 26% (p=0.96), respectively.

A total of 493 infectious episodes were observed up to day +100 after transplant, of which 286 were in Cohort A and 207 were in Cohort B. Overall, the percentage of patients with at least one infection event by day +100 remained stable over the two time periods (82% vs. 87%; p=0.34). The distribution of infections with organ localization (including fungal infections), BSIs, and viral reactivations in Cohort A vs. Cohort B was: 40% vs. 44% (p=0.45), 23% vs. 26% (p=0.34), and 37% vs. 30% (p=0.12).

Between day +100 and day +180, 120 infectious events occurred, of which 67 were in Cohort A and 53 were in Cohort B. In Cohort A and Cohort B, 37% and 27% of the patients developed at least one infection event between day +100 and +180 (p=0.15), respectively. The distribution of infections with organ localization (including fungal infections), BSIs, and viral reactivations in Cohort A vs. Cohort B was: 42% vs. 40% (p=0.81), 18% vs. 11% (p=0.32) and 40% vs. 49% (p=0.12).

### Infections With Organ Localization

**Figure 1:**
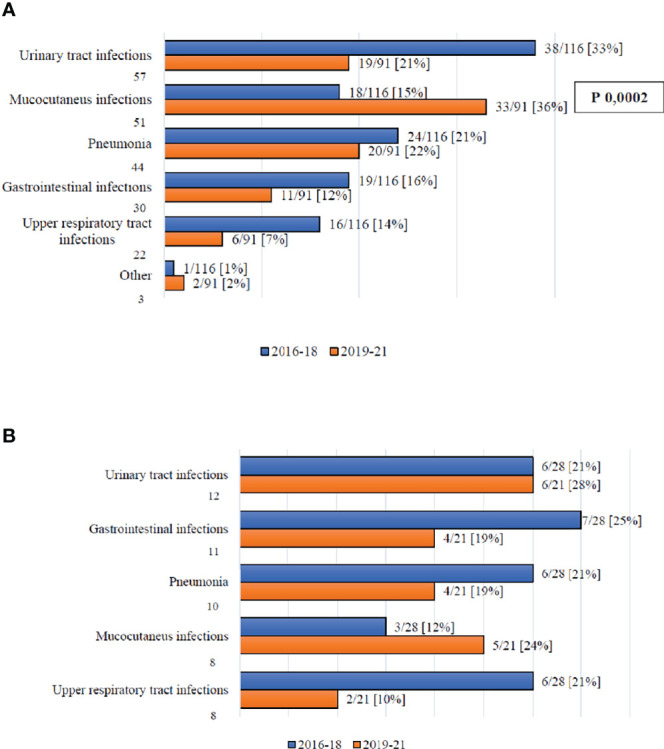
**(A)** Infection complications with organ localization by day +100. **(B)** Infection complications with organ localization between day +100 and +180.

**Figure 1** reports the organ involvement in the case of infections with organ localizations by day +100 (1A) and between day +100 and day +180 (1B). The only significant difference is with regards to the incidence of mucocutaneous infections, which increased moving from Cohort A to Cohort B (15% vs. 36%, p=0.0002).

**Figure 2:**
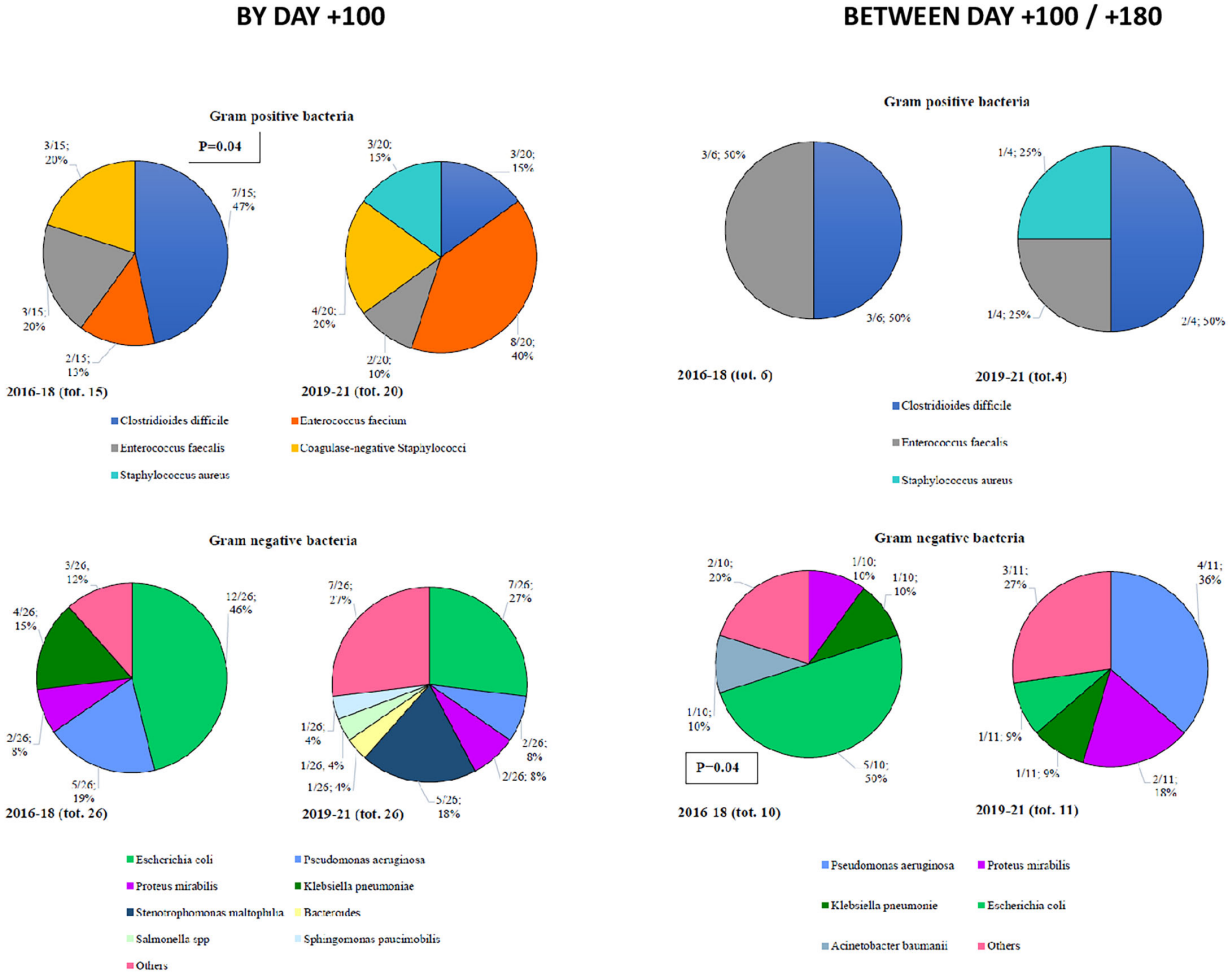
Bacterial isolates in infectious complications with organ localization after allo-SCT.

By day +100, 35 Gram-positive bacteria (15 in Cohort A and 20 in Cohort B) and 52 Gram-negative bacteria (26 in each of the two Cohorts) were isolated (**Figure 2**). Among Gram-positive bacteria, the most frequent isolation was *Clostridiodes difficile* (47%), coagulase negative *Stafilococci* (20%), and *E faecalis* (20%) in Cohort A and *E faecium* (40%), coagulase negative *Stafilococci* (20%), *Clostridiodes difficile* (15%), and *S aureus* (15%) in Cohort B. Notably, we observed a statistically significant reduction of *Clostridioides difficile* moving from Cohort A to Cohort B (47% vs. 15%, p=0.04). Among Gram-negative bacteria, *E Coli* (46%) and *P aeruginosa* (19%) in Cohort A, and *E. coli* (27%) and *S maltophilia* (18%) in Cohort B were more frequently isolated (**Figure 2**).

In terms of late infections (day +100 to day +180), 10 Gram-positive (6 in Cohort A and 4 in Cohort B) and 21 Gram-negative (10 in Cohort A and 11 in Cohort B) bacteria were recorded (**Figure 2**). Among Gram-positive bacteria, 50% of the isolates were *Clostridiodes difficile*, in both Cohorts. Focusing on Gram-negative bacteria, *E. coli* in Cohort A (50%) and *P aeruginosa* in Cohort B (36%) were more frequently isolated (**Figure 2**).

Moreover, we collected data on viral and fungal isolates with organ localization at day +100 and between day +100 and +180 (**Supplementary Figure S1**). No significant differences were observed between the two Cohorts. *BK-polioma virus* (Cohort A) and *herpes simplex virus 1* (Cohort B) were the most frequently isolated at both time points, respectively. Among fungi, *Aspergillus spp* was prevalent in both Cohorts, at both time points.

Finally, we compared the overall survival (OS) of the two Cohorts according to the development of infection with organ localization, independently from the pathogen. Comparing Cohort A and Cohort B by day +100, we observed a 2-year OS (event vs. no event) of 42.2% vs. 65.4% (p=0.011) and 46.2% vs. 56.7% (p=0.76), respectively (figure not shown). Focusing on OS with regard to late infections with organ localizations we registered a 2-year OS (event vs. No event) of 52.4% vs. 75.4% in Cohort A (p=0.41) and 75% vs. 70% (p=0.41) in Cohort B (figure not shown).

### Bloodstream Infections (BSIs)

By day +30, 34/116 (29%) and 34/84 (40%) patients developed a BSI in Cohort A and B, respectively (p=0.09), for a total of 37 and 42 BSIs (**Table 3**). Focusing on Gram-negative BSIs, 14 episodes were observed in 14/116 patients (12%) in Cohort A(and 22 episodes were observed in 19/84 patients (23%) in Cohort B (p= 0.05). The distribution of the different species was comparable, with E. *coli* and K *pneumoniae* the most frequent isolates. Regarding Gram-positive BSIs, 21 episodes were recorded in 20/116 patients (17%) in Cohort A, and 16 episodes were recorded in 14/84 patients (17%) in Cohort B (p=0,91). Coagulase negative *Stafilococci* were most frequently isolated, with no significant difference between the two Cohorts. The remaining episodes in each Cohort were polymicrobial (two in Cohort A and four in Cohort B).

Moving to day +100, a total of 119 BSIs were observed: 65 (55%) in Cohort A and 54 (45%) in Cohort B. Late BSIs (day +100 to +180) numbered 18: 12 (67%) in Cohort A and 6 (33%) in Cohort B (**Supplementary Figure S2**). No differences were observed in terms of Gram-positive vs. Gram-negative distribution, nor in terms of species. Regarding early BSIs, coagulase-negative *Stafilococci* and *E. coli* were the most frequent isolates in both Cohorts (**Supplementary Figure S3**).

The antibiotic resistance of *E. coli*, K *pneumoniae*, and P *aeruginosa* isolated in all the BSIs is reported in **Table 4**. In particular, comparing Cohort A and Cohort B, fluoroquinolone resistance was the most frequently observed (60% vs. 47%; p=0.25) with a trend of significant reduction in the case of K *pneumoniae* (78% vs. 33%; p=0.05). ESBL+ bacteria and CRE were isolated in 21% vs. 17% (p=0.63) and 5% vs. 8% (p=0.6), respectively, whereas MDRO were found in 5% vs. 8% of the cases (p=0.60).

**Table 4:** Antibiotic resistance of E. *coli*, K *pneumoniae*, and P *aeruginosa* isolated in all the BSIs.

Bacteria species	Cohort A (n = 38)		Cohort B (n = 36)		P-value
	N°	%	N°	%	
**E. *coli***	**15**	**39**	**10**	**28**	**0.29**
- ESBL+ - CRE - Fluoro quinolone-R - MDRO	6 0 14 0	40 0 93 0	3 0 7 0	3 0 0 70 0	0,61 - 0,12 -
**K *penumoniae***	**9**	**24**	**9**	**25**	**0,89**
- ESBL+ - CRE - Fluoro quinolone-R - MDRO	2 2 7 2	22 22 78 22	3 2 3 2	33 22 33 22	0,6 1 **0,05** 1
**P a*eruginosa***	**5**	**13**	**9**	**25**	**0,19**
- Fluoro quinolone-R - MDRO	0 0	0 0	3 0	33 0	- -
**TOTAL**	**38**	**-**	**36**	**-**	**-**
- ESBL+ - CRE - Fluoro quinolone-R - MDRO	8 2 23 2	21 5 60 5	6 3 17 3	17 8 47 8	0,63 0,60 0,25 0,60

BSIs, blood-stream infections; ESBL+, extended spectrum beta-lactamases producers; CRE, Carbapenem-Resistant Entrobactriaceae; MDRO, MultiDrug ResistantOrganism.

Bold values regard the statistical significance (when below 0.05).

**Figure 3:**
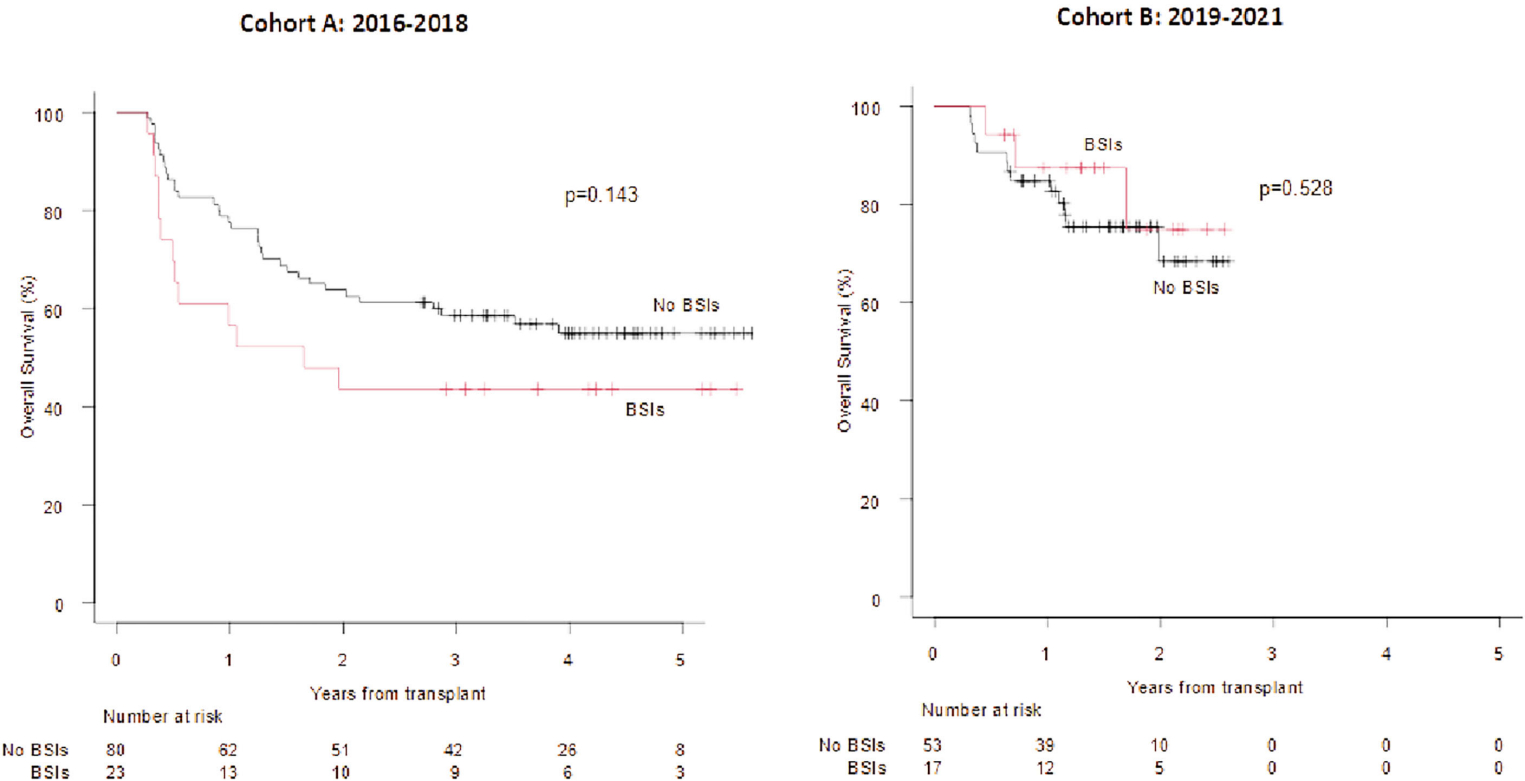
OS according to the development of BSIs from Gram negative bacteria (+100 days Landmark analysis).OS at 1 and 2 years in case of BSIs from Gram negative bacteria vs no event (cohort A) 63.8% vs 77.5% and 43.5% vs 56.5%. OS at 1 and 2 years in case of BSIs from Gram negative bacteria vs no event (cohort B): 87.4% vs 84.9% and 74.9% vs 68.5%.

The OS was not affected by the development of a BSI, neither by day +100 nor between day +100 and +180, even though a more favourable trend was observed for patients who did not develop a BSI in both Cohorts (figure not shown). Interestingly, the 2 year OS of patients who developed a Gram-negative BSI by day +100, vs. those without an event was 43.5% vs. 56.5% in Cohort A (p=0.143) and 74.9% vs. 68.5% (p=0.528) in Cohort B (**Figure 3**; day +100 Landmark analysis).

### Incidence and Distribution of Fungal and Viral Infections

**Figure 4:**
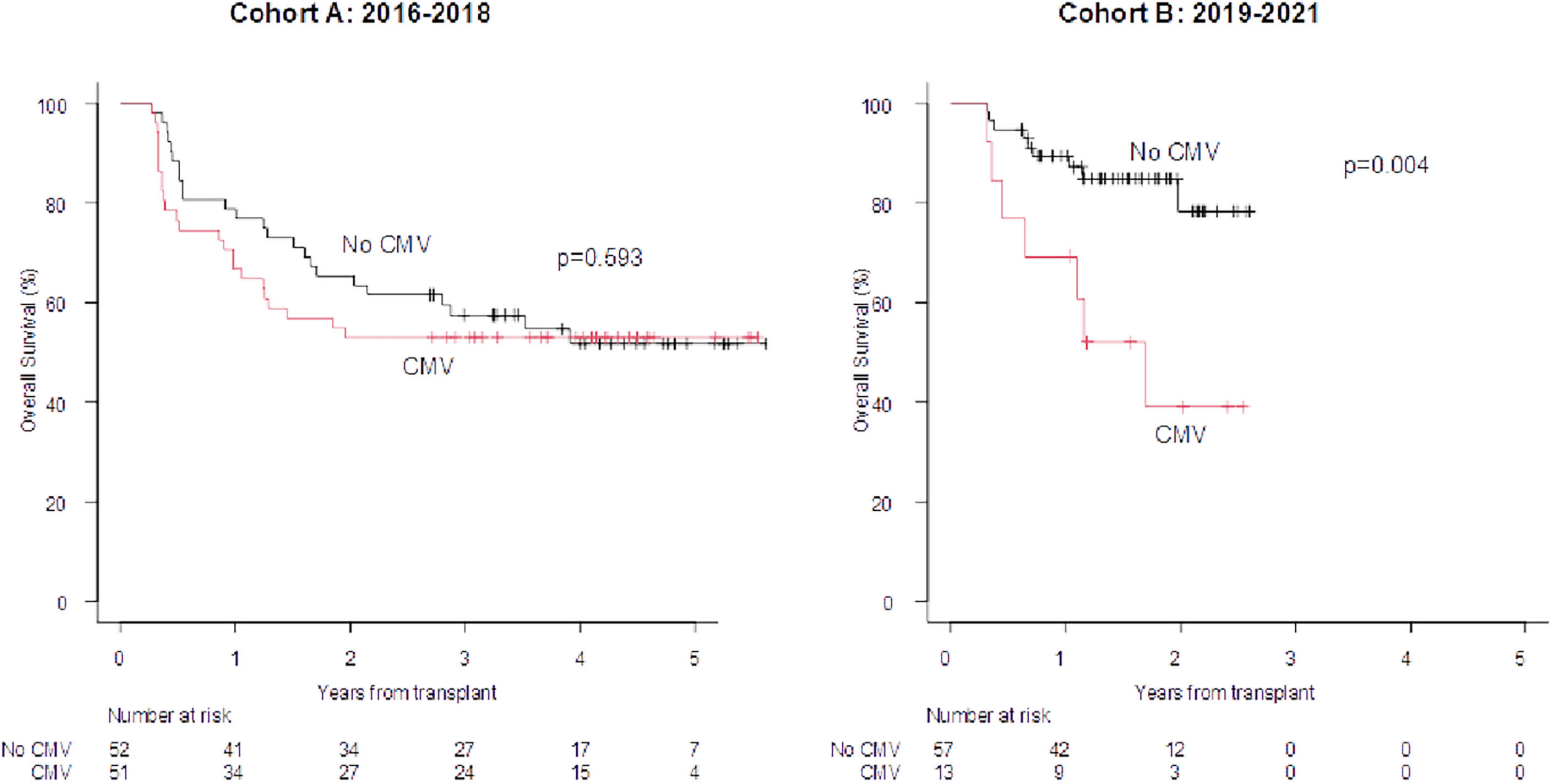
100-days Landmark OS according to the development of CMV reactivation by day +100 (any level of DNA). OS at 1 and 2 years in case of CMV infections at +100 days vs no event (cohort A): 66.7% 78.8% and 52.9% vs 65.4%. OS at 1 and 2 years in case of CMV infection at +100 days vs no event (cohort B): 69.2% vs 89.3% and 38.9% vs 78.3%.

The overall incidence of proven-probable invasive fungal infections (IFIs) was comparable in the two Cohorts (13% vs. 18% in Cohort A and B, respectively) (**Supplementary Figure S4A**). By day +100, the incidence of CMV reactivations (any level of DNA) was significantly reduced in Cohort B (51% vs. 15%; p=0.00001), whereas the incidence of herpes virus 6 reactivations significantly increased (4% vs. 14%, p=0.00001) (**Supplementary Figure S4B**). Late CMV reactivations (any level of DNA between day +100 and day +180) were comparable in the two Cohorts (47% vs. 42%) (**Supplementary Figure S4C**). The OS was not affected by the development of a fungal infection in either Cohorts. Similarly, the development of viral infection both by day +100 and between day +100 and day +180 did not impair the outcome in either Cohort A or in Cohort B (figure not shown). Interestingly, restricting the analysis on CMV reactivations (any level of DNA) by day +100, the OS was significantly impaired in Cohort B only (2019–2021) (2 year OS 38.9% vs. 78.3%; p=0.004; **Figure 4**; day +100 Landmark analysis).

## Discussion

The long-term outcome of patients submitted to allo-SCT is strongly influenced by two events: disease recurrence and non-relapse mortality (NRM). These events are responsible for the failure of the transplant procedure, which happens in approximately 50% of all transplants (8). One way to reduce NRM is to reduce the incidence and the mortality of infectious complications, which are recorded in more than two-thirds of patients submitted to allo-SCT (1). The management of infections is complex, and involves hematologists as well as specialists in infectious diseases, and microbiologists. This management includes prophylaxis and treatment programs that need to be frequently revised and updated, according to the monitoring of local epidemiology and changes in transplant platforms.

In 2017, we published the results of microbiological monitoring conducted in our centre from 2010 to 2015 (7). We focused on BSIs that occurred in 162 allotransplanted patients. Approximately half of the patients experienced a BSI that was sustained by a Gram-positive bacteria in 65% of cases (namely, coagulase-negative *Staphilococci*). Among Gram-negative bacteria, *E. coli* and *P aeruginosa* were more frequently isolated. Fluoroquinolone resistance was observed in almost all the isolates, 76% of *E. coli* were extended-spectrum beta-lactamase producers (ESBL+), and 40% of *P aeruginosa* were carbapenem-resistant. In response to these data, we decided to modify our program of infectious prophylaxis and treatment, according to the BATMO protocol, which has been in use in our centre since 2019 (**Table 1**). In the same period of time, a strong and intense antimicrobial stewardship has been implemented in our hospital, with the aim to significantly reduce the use of antibiotics and, hopefully, the percentage of MDRO.

In this analysis, we compared the distribution and outcome of infectious complications recorded between 2019 and 2021 (Cohort B), with those registered before BATMO (2016-2018; Cohort A), collecting data on infections with organ localizations (including fungal infections), BSIs, and viral infections. It is important to highlight that the management of infections during pre-transplant therapeutic phases (e.g., induction and consolidation for acute leukemias, autologous SCT) did not change in the two time periods. Analyzing the distribution of all types of infections both by day +30 and +100, and between day +100 and +180, the only significant difference found was the number of patients with at least one infectious event at day +30 (60% vs. 80% in Cohort A and B, respectively; p=0.003) (**Table 3**). Following a more detailed analysis of infections with organ localizations, it is worth highlighting that mucocutaneous infections were more frequent in Cohort B by day +100 (15% vs. 36%, p=0.0002; **Figure 1**). This may be related to more frequent use of myeloablative conditioning regimens, with an expected higher mucosal toxicity (**Table 2**) (17 – 17). Moreover, a statistically significant reduction of *Clostridioides difficile* by day +100 was observed moving from Cohort A to Cohort B (47% vs. 15%, p=0.04; **Figure 2**). This can be considered a direct effect of the abolishment of fluoroquinolone prophylaxis, as it is well known that chronic antibiosis induces a disruption of intestinal microbiota, which favours *Clostridiodes* infections (18–20). Overall, *Clostridiodes* are still involved in a high percentage of infections, also in Cohort B. This can be related to an increased number of myeloablative conditioning regimens, and haploidentical donors, in this Cohort (**Table 3**). In both these situations, a higher gastrointestinal toxicity and a prolonged use of antibiotics are expected, and these conditions may alter the homeostasis of intestinal microbiota, thus favouring *Clostridiodes* infections.

With regards to early and late BSIs, the distribution of Gram-positive vs. Gram-negative isolates was superimposable in both Cohorts, with a prevalence of coagulase-negative *Stafilococci* and *E. coli* in both Cohorts (**Supplementary Figure S3**). Notably, the incidence of BSIs at day +30 sustained by Gram-negative bacteria was 12% vs. 23% in Cohort A vs. Cohort B, respectively (p=0.05). Even though the lack of a Gram-negative directed prophylaxis in Cohort B may partially be responsible for this finding, another possible explanation is represented by the differences between the two Cohorts: more myeloablative conditionings and more haploidentical transplants are present in Cohort B, suggesting a higher risk for Gram-negative BSIs. Considering antibiotic resistance of the most frequent Gram-negative isolates irrespective of the time point of detection (*E. coli, K pneumoniae, and P aeruginosa*) we observed that fluoroquinolone resistance is still prevalent, though a trend towards a reduction moving from Cohort A to Cohort B was observed, in particular for *K pneumoniae* (78% vs. 33%; p=0.05). MDRO isolates remained stable over time (5% vs. 8%; p=0.60). The stability of the profile of antibiotic resistance over time probably reflects the fact that the prophylaxis and treatment of pre-transplant bacterial infections (e.g., during induction/consolidation for acute leukemias or following autologous SCT) remained unchanged, as previously reported. On the other hand, the progressive reduction of fluoroquinolone resistance, ESBL+ Gram negative bacteria and CRE from 2010 (7) to 2019 may be considered the result of the intensive policy of antimicrobial stewardship implemented in our hospital over time.

Focusing on mortality due to infections, 13/116 patients (11%) and 8/84 patients (9%) in Cohort A and B died due to infectious complications, respectively (p= 0.70). More in detail, 2/13 (15%) and 2/8 (25%) deaths in Cohort A and B were caused by Gram-negative BSIs, respectively (p=0.29). Interestingly, even though the percentage of patients with pre-transplant colonization was higher in Cohort B (11% vs. 26%; p=0.005; **Table 3**), this did not translate into a significantly higher incidence of Gram-negative BSIs and significantly higher mortality due to Gram-negative bacteremia. Finally, focusing on the cause of death by day +30, 1/116 (1%), and 3/84 (4%) patients died due to infectious complications, in Cohort A and Cohort B, respectively (p= 0.17).Looking at the long-term outcome, the OS of patients who developed a BSI sustained by a Gram-negative bacteria by day +100 vs. those who did not was comparable in the two Cohorts (**Figure 3**). Notably, 5/19 (26%) patients who had a Gram-negative BSI in Cohort B received first-line treatment with new antibiotics (e.g., ceftazolan-tazobactam), based on pre-transplant microbiological history and/or colonization. The high efficacy of these antibiotics may partially explain the similar outcome of patients in both Cohort A and B, despite a higher incidence of Gram-negative BSIs in Cohort B. This suggests that fluoroquinolone can be safely avoided during the neutropenic phase. As described by Mikulska and colleagues (21), fluoroquinolone prophylaxis during neutropenia did not have effects on mortality but was associated with a lower rate of BSIs and episodes of fever during neutropenia. In a few studies, fluoroquinolone prophylaxis has resulted in an increase in colonisation or infection with fluoroquinolone, or multi-drug resistant strains (22).

Regarding fungal infections, the major finding of this analysis is that the incidence of proven/probable invasive fungal infections remained stable moving from Cohort A to Cohort B (**Supplementary Figure S2A**), even though the proportion of patients at risk of this complication increased (more myeloablative conditioning regimens and more haploidentical transplants in Cohort B; **Table 2**) (4, 17, 23–25). This observation is of interest and may be related to the intensification of fungal prophylaxis in high-risk patients according to the BATMO protocol, with posaconazole instead of fluconazole (**Table 1**), in accordance with the risk score proposed by Stanzani et al. (13). Finally, focusing on viral infections, the impact of CMV reactivations by day +100 (any level of DNA) significantly reduced moving from Cohort A to Cohort B (51% in Cohort A vs. 15% in Cohort B; p=0.00001; **Supplementary Figure S4B**), and this is in line with our recently published study (26). This reflects the efficacy of letermovir prophylaxis, but the issue of CMV is not completely resolved, as the OS of patients with a CMV reactivation is significantly impaired in comparison to those without this event (**Supplementary Figure 4B**). This suggests that CMV still affects the outcome, even in the letermovir era. Interestingly, of the 25 episodes of CMV reactivations by day +100 in Cohort B, 17 were considered clinically significant. Sixteen out of these 17 clinically significant episodes occurred during aGVHD, and this may explain the impaired OS registered in this subset of patients. Moreover, as reported in **Supplementary Figure 4C**, the number of CMV reactivations beyond day +100 (any level of DNA), when letermovir was discontinued, rises up to 42%, equal to the 47% observed in Cohort A, when letermovir was not available. Concerning this latter aspect, the results of the registrative trial of letermovir day +100/+200 are highly awaited. The other virus that modified its impact was herpes virus 6 (HHV6): its incidence moved from 4% in Cohort A to 14% in Cohort B (p=0.00001; **Supplementary Figure 4B**). This is in line with the more frequent use of haploidentical donors in Cohort B, as it is known that haploidentical transplants are associated with a higher incidence of HHV6 reactivations (27).

Nearly 2 years after the adoption of the BATMO protocol, we can conclude that: i) fluoroquinolone prophylaxis during the neutropenic phase can be safely abolished; ii) the intensification of fungal prophylaxis in high-risk patients with a highly anti-mould drug such as posaconazole is a good choice, particularly in the era of haploidentical transplants; iii) letermovir significantly reduced the clinical impact of CMV reactivations by day +100. In summary, BATMO protocol is an example of a specific antimicrobial therapy that was developed at our centre to improve infection treatments for patients submitted to allo-SCT. We are aware that the efficacy and safety of this protocol are strictly dependent on the ecology of our transplant centre and that similar results may not be observed in other centres with different microbiological epidemiology. Overall, we consider BATMO protocol the result of an active antimicrobial stewardship that will be modified in the future and adapted to local epidemiology, new anti-infectious drugs, and new conditioning platforms for allo-SCT.

## Data Availability Statement

The raw data supporting the conclusions of this article will be made available by the authors, without undue reservation.

## Ethics Statement

Ethical review and approval was not required for the study on human participants in accordance with the local legislation and institutional requirements. The patients/participants provided their written informed consent to participate in this study.

## Author Contributions

MM, AT, and LM collected the data; MM, AT, LM, and NP analyzed the data; MM, AT, LS, NP, and DR wrote the manuscript. All the authors revised the manuscript and gave their approval before submission.

## Conflict of Interest

The authors declare that the research was conducted in the absence of any commercial or financial relationships that could be construed as a potential conflict of interest.

## Publisher’s Note

All claims expressed in this article are solely those of the authors and do not necessarily represent those of their affiliated organizations, or those of the publisher, the editors and the reviewers. Any product that may be evaluated in this article, or claim that may be made by its manufacturer, is not guaranteed or endorsed by the publisher.
