# Supplementary figures and images for: Results of an Innovative Program for Surveillance, Prophylaxis, and Treatment of Infectious Complications Following Allogeneic Stem Cell Transplantation in Hematological Malignancies (BATMO Protocol)
Source: Front Oncol. 2022 Jun 17;12:874117. doi: 10.3389/fonc.2022.874117 (PMC9247274; doi:10.3389/fonc.2022.874117)

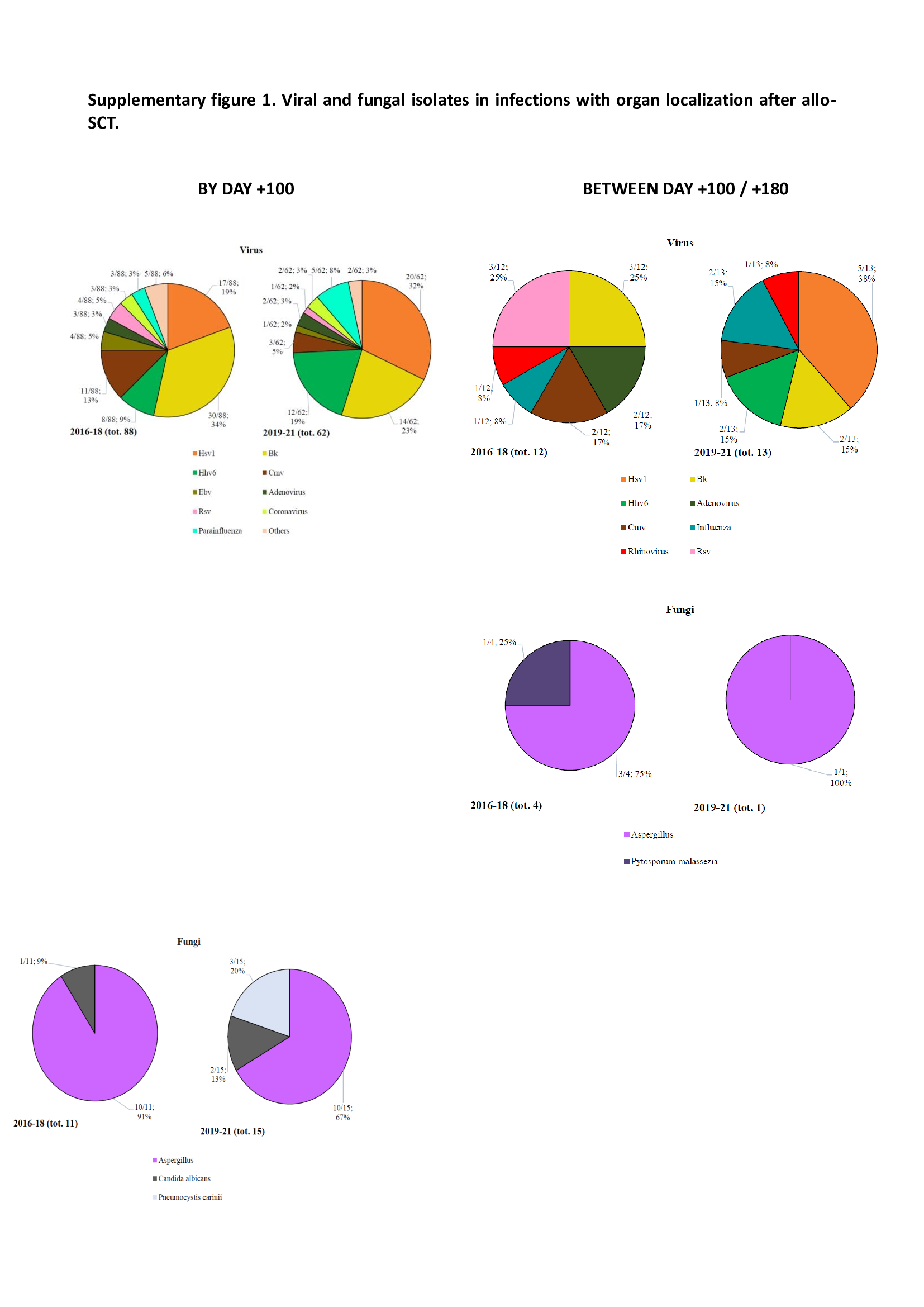

Supplement: Supplementary file 1 [file Image_1.jpeg]

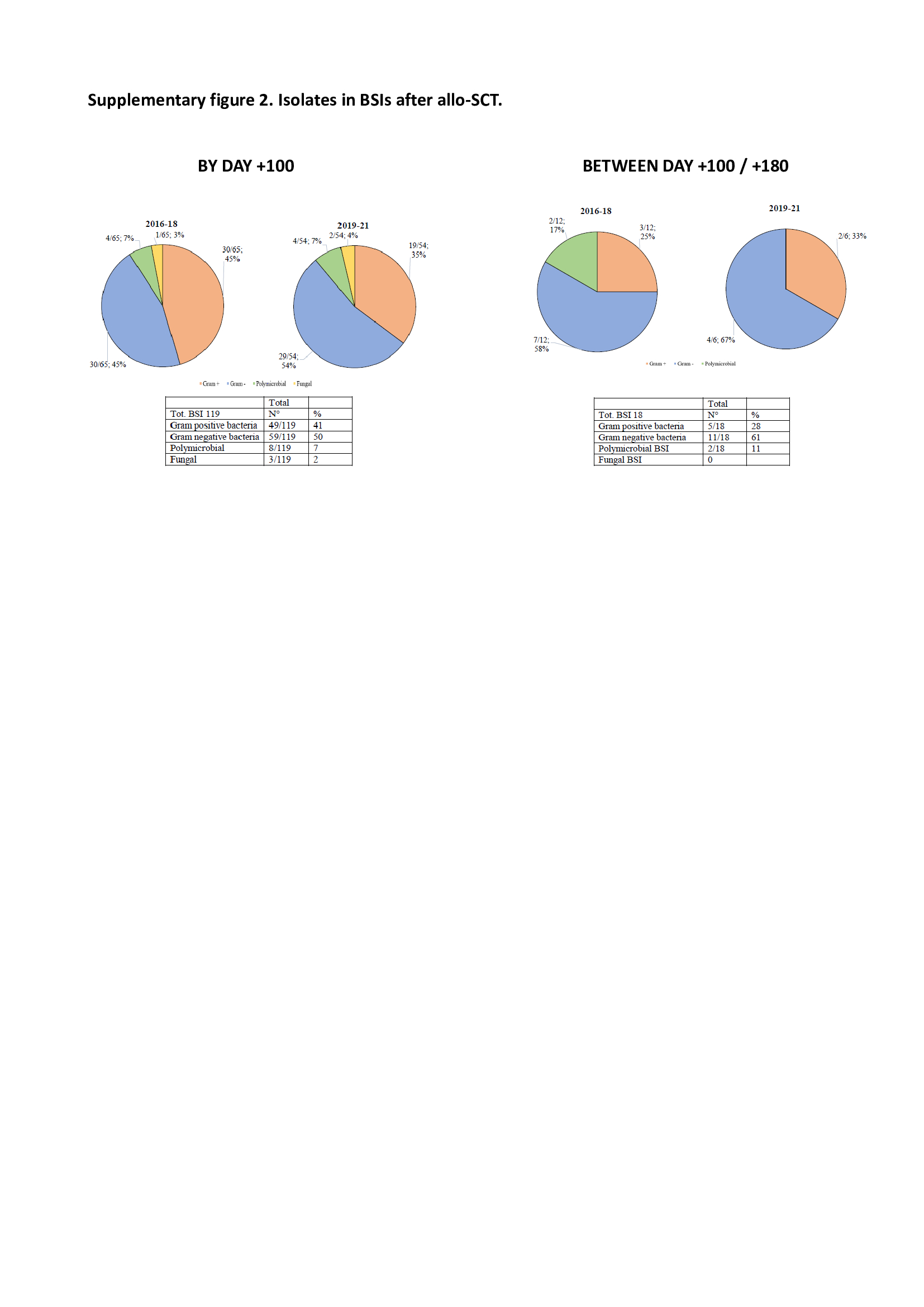

Supplement: Supplementary file 2 [file Image_2.jpeg]

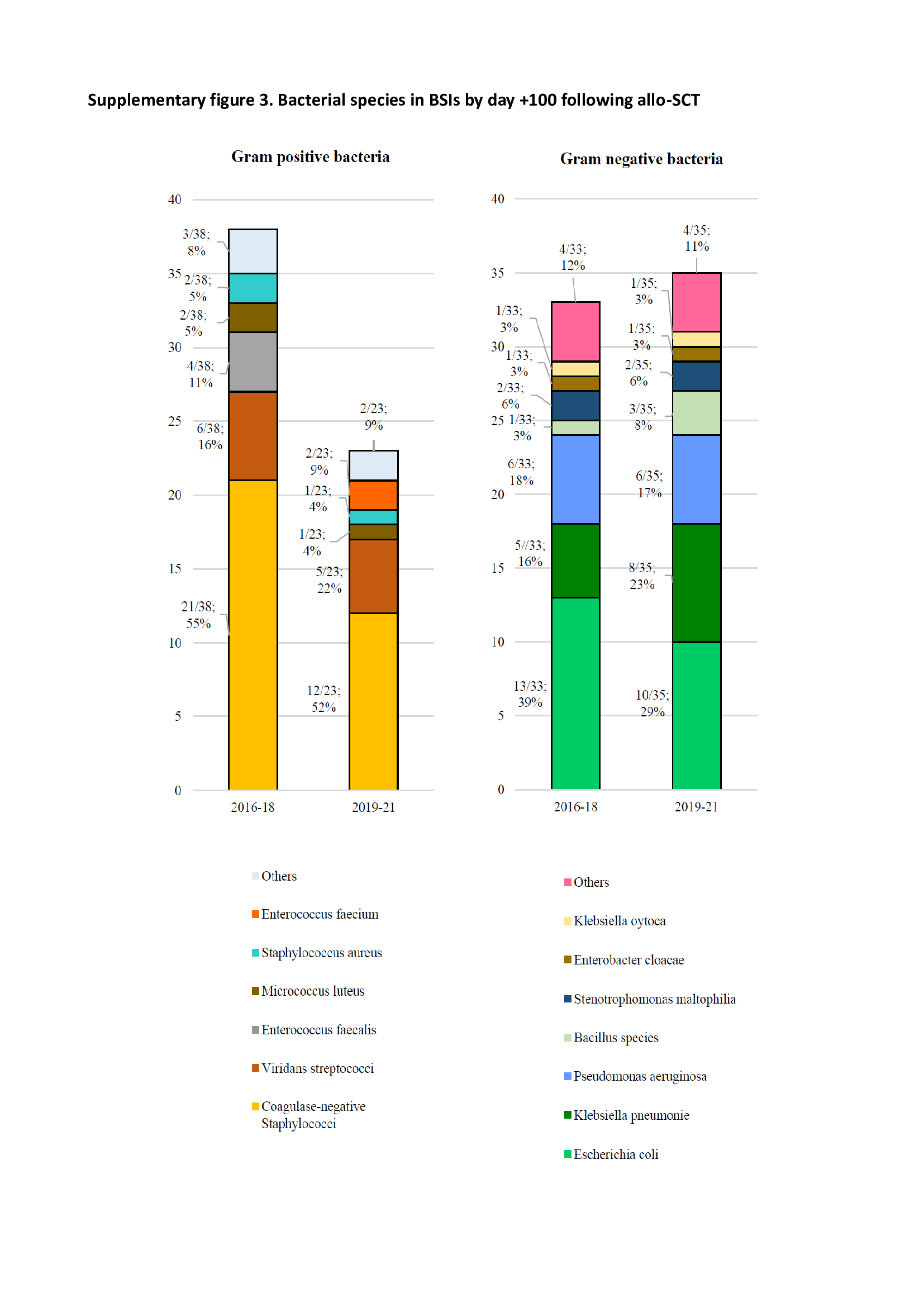

Supplement: Supplementary file 3 [file Image_3.jpeg]

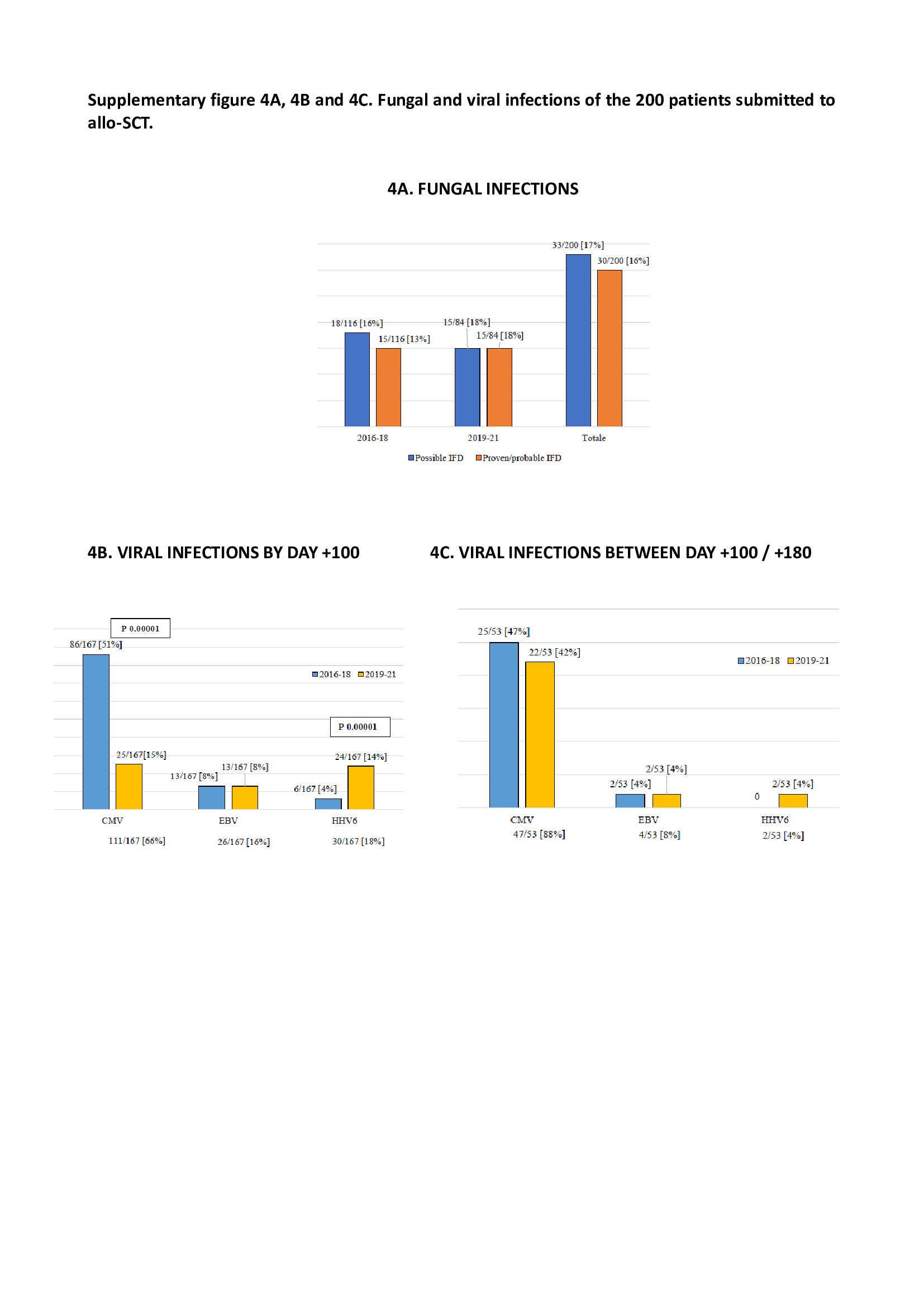

Supplement: Supplementary file 4 [file Image_4.jpeg]
